# Asthma in Black African, Black Caribbean and South Asian adolescents in the MRC DASH study: a cross sectional analysis

**DOI:** 10.1186/1471-2431-10-18

**Published:** 2010-03-25

**Authors:** Melissa J Whitrow, Seeromanie Harding

**Affiliations:** 1 Social and Public Health Sciences Unit, Medical Research Council, Glasgow, UK

## Abstract

**Background:**

Ethnic differences in the prevalence of asthma among children in the UK are under-researched. We aimed to determine the ethnic differences in the prevalence of asthma and atopic asthma in children from the main UK ethnic groups, and whether differences are associated with differential distributions in social and psychosocial risk factors.

**Methods:**

6,643 pupils aged 11-13 years, 80% ethnic minorities. Outcomes were asthma/wheeze with (atopic) and without hay fever/eczema. Risk factors examined were family history of asthma, length of residence in the UK, socioeconomic disadvantage, tobacco exposure, psychological well-being, and body mass index (BMI).

**Results:**

There was a pattern of lower prevalence of asthma in Black African boys and girls, and Indian and Bangladeshi girls compared to White UK. The overall prevalence was higher in Mixed Black Caribbean/White boys, with more atopic asthma in Black Caribbean boys and Mixed Black Caribbean/White boys due to more hayfever. Poor psychological well-being and family history of asthma were associated with an increased risk of asthma within each ethnic group. UK residence for ≤ 5 years was protective for Black Caribbeans and Black Africans. Increased BMI was associated with an increased reporting of asthma for Black Africans. Adjustments for all variables did not remove the excess asthma reported by Black Caribbean boys (atopic) or Mixed Black Caribbean/White boys.

**Conclusion:**

The protective effect of being born abroad accounted for ethnic differences in some groups, signalling a role for socio-environmental factors in patterning ethnic differences in asthma in adolescence.

## Background

Studies of ethnic minority children living in the United Kingdom (UK) and United States (US) have reported inequalities in the prevalence of asthma, wheeze and atopy [1-5]. In the Health Survey for England (HSE) Black African and South Asian (Indian, Pakistani or Bangladeshi) children (< 16 yrs) were less likely to have been diagnosed with asthma by a doctor than the general population[6]. At 3 years, Black Caribbean children have higher risk, and Bangladeshis lower risk of asthma compared to White UK, linked to socio-economic and cultural factors[7]. In the US, young (≤ 3 yrs) African American children have excess asthma and respiratory illness compared to Whites[8,9]. Studies of asthma in very young children (< 3 yrs) are likely to include those with transient wheeze that might not persist later in childhood. Currently there are no UK studies reporting and explaining ethnic differences in asthma in late childhood (11-13 yrs).

Socio-environmental factors may influence risk of asthma and atopy through early life exposures (i.e. respiratory infection, endotoxin, parasites) regulating the allergic inflammatory response and/or later life exposures to allergens (i.e. pollution, animals)[10,11]. A positive association between body mass index (BMI) and asthma has been reported, possibly acting via a mechanical process or the inflammation or sympathetic nervous system[12], however evidence for a causal relationship is inconsistent[13]. The authors have previously reported greater BMI in Black Caribbean and Black African girls compared with their white British peers[14], hence BMI may influence asthma prevalence in these groups. Asthma has a strong hereditary component; children with one or more first degree relatives with asthma have at least a fifty percent increase in risk of asthma themselves compared to children with no family history[15]. Genetic studies have identified a number of polymorphisms potentially associated with a predisposition to asthma and atopy; but the relationship between polymorphisms and environmental exposures is poorly understood[16].

The International Study of Asthma and Allergy in Childhood (ISAAC) found large between country variation in asthma and allergy, with more westernised countries (i.e. the UK) having greater prevalence than those less developed[17]. Children born in developing countries who migrate to the UK at early ages are likely to have a similar genetic predisposition to asthma to children of the same ethnicity born in the UK. Potentially different environmental exposures in the latter group may moderate the protective risk[18]. Studies looking at asthma variation within ethnic minority groups in Western countries are few[18], but these point to the importance of time since, or age at, migration on asthma prevalence[19,20], and hence potentially critical periods for protective exposures in home countries or adverse exposures in new countries.

The objectives of this paper are (1) To determine the ethnic differences in the prevalence of asthma and atopic asthma in children from the main ethnic groups in the UK (White UK, Black African, Black Caribbean, Indian, Pakistani, Bangladeshi and Mixed Black Caribbean/White UK); (2) To identify the social and psychosocial risk factors for asthma and atopic asthma in children in each group and (3) To determine whether ethnic differences in asthma and atopic asthma are associated with differential distributions in risk factors. To do this we use the MRC DASH (Determinants of Adolescent Social well-being and Health) study, a sample of children from London schools which contains a range measures on potential risk factors (e.g. socio-economic disadvantage, migration status, family history of asthma, parental smoking, body size). Preliminary results from this work have been previously reported in the form of an abstract[21].

## Methods

### Design and sample

The DASH study has been described previously[22]. Briefly, the sample was recruited from 51 schools in ten inner London boroughs with high proportions of the main ethnic minority groups. Pupils from Years 7 and 8 (aged 11-13 years) in randomly selected mixed ability classes were invited to join the study. Approvals from the Multi-centre Research Ethics Committee and from Local Education Authorities were obtained. Active (opt-in) consent was used for pupils and passive (opt-out) consent for parents. The pupil response rate was 81%.

Ethnicity of White UK, Black Caribbean, Black African, Indian, Pakistani and Bangladeshi origin was self defined. Pupils who reported 'Black British' or 'Asian British' or who did not report their own ethnicity were classified using reported parental ethnicity and parental and grandparental country of birth using a rule of having at least one parent with an ethnicity reflecting home countries of grandparents and having at least three grandparents who were born in the home countries. Among those reporting Mixed ethnicities, Mixed White and Black Caribbean pupils were included in these analyses as they were the only sizeable group. First generation was defined as born abroad versus born in the UK. Among pupils born abroad we distinguished between ≤ 5 years and > 5 years residence in the UK. These migration variables were used as a proxy measure of the extent of early life environmental influences in the UK. In addition we also examined having at least one parent born abroad and the number of grandparents born abroad. The rational for this being that the transmission of asthma risk across generations may be influenced by the extent to which parents and grandparents were exposed to protective or adverse environments in home countries.

In a total sample of 6643 pupils, 6465 (97%) self reported their asthma status and did not have cystic fibrosis. The final sample for analysis comprised of 1219 White UK, 933 Black Caribbean, 1095 Black African, 459 Indian, 215 Pakistani, 392 Bangladeshi and 299 Mixed White UK and Black Caribbean pupils. Other mixed ethnicity (n = 262), and other ethnicity (n = 1591) pupils were excluded from the analysis as they consisted of a number of small ethnic minority groups (e.g. East Asians, Eastern Europeans, Afghanis, North African Arabs).

### Outcome measures and potential risk factors

Affirmative asthma status was determined by the child reporting ever having asthma ("Have you ever had asthma?", n = 659), recent breathing difficulties or wheeze ("In the last month, have you had breathing difficulties or wheeze?", n = 672), or both (n = 508). In the absence of skin prick or blood test for atopy, asthma was defined as atopic if accompanied by one or more indicators of allergy (hay fever, n = 522; eczema or skin allergies, n = 193; both n = 217). Children also reported their family history of asthma (maternal, paternal, one or more grandparent with asthma).

Measuring socio-economic status (SES) among minority groups is complex and a multi dimensional index appears to be more discriminating of health differences[23,24]. It is also problematic to obtain some information (e.g. parental occupation) from children. With this in mind, SES was measured using 17 standard of living items (for example family car, television and computer ownership, expressed as tertiles), parental employment status (employed, unemployed, other), and family type (lone versus dual parent household). Exposure to passive cigarette smoking was also examined, affirmed if the pupil reported living with a parent who currently smokes. The potential influence of childhood infection, in utero programming or endocrine effects was examined using number of siblings (none, > 1)[25]. Psychological well-being was measured using the mean total difficulties score (TDS) from the Goodman's Strengths and Difficulties Questionnaire (see http://www.sdqinfo.com/). Increasing TDS reflects increasingly poorer psychological well-being[26]. Standardised measures were taken of height, using portable stadiometers, and weight, using Salter electronic scales. Body Mass Index (BMI) was derived as weight (kg)/height (m^2^). BMI was converted to standard deviation scores and a continuous percentile variable based on the 1990 British growth reference curves[27].

### Model building

Multiple logistic regression models were used to examine the risk factors for asthma and atopic asthma within and between ethnic groups. Firstly, each of the potential risk factors outlined above were examined separately within each ethnic group. Secondly, differences between ethnic groups were investigated in a stepwise model with ethnicity as the main explanatory variable. To disentangle the effects of ethnicity, potential risk factors for asthma that are known to vary by ethnicity were then included in the model as follows. Measures of SES (Model 2), generational status (M3), familial asthma (M4), parental smoking (M5), psychological well-being (M6) and body size (M7) were cumulatively added to successive models in a stepwise model building process (i.e. M7 contained all of the variables). The order of adding these variables did not have an impact on the results. Primary and secondary interactions were investigated in all models. All models were then rerun for atopic asthma (non asthmatics as baseline). All models were adjusted for sex and age. The effect of clustering in schools on standard errors and confidence intervals was accounted for in all models using the xtlogit command in Stata with the random effect option.

## Results

Black Africans, Indians and Bangladeshis had a lower prevalence of asthma, and Mixed White/Black Caribbeans had a higher prevalence than White UK children (Additional file 1: Table S1). Black Caribbean and Mixed White/Black Caribbean asthmatics were more likely to be atopic than other ethnic groups; primarily due to an excess of hay fever. Black Caribbean and Black African children were less likely to report a positive family history (parent or grandparent) of asthma than White UK, however there was a higher proportion of inadequately described family history of asthma in this group.

### Asthma risk factors within each ethnic group

Additional file 1: Table S2 shows that TDS (with the exception of Pakistanis) and having at least one parent with asthma (with the exception of Mixed White/Black Caribbean group) were independent risk factors for asthma within groups. In White UK, Black Caribbean and Black Africans, grandparental asthma was also associated with increased asthma risk. Other distinguishing features for specific groups include less reported asthma in Black Caribbeans and Black Africans who had spent ≤ 5 years in the UK compared with those born in the UK, less asthma in the least advantaged tertile of SES compared with those in the most advantaged, greater risk with increasing BMI in Black Africans, and more reported asthma if a parent smoked in the household in Black Caribbeans. A gender difference was observed for Indians and Bangladeshis, with less asthma in girls than boys.

The independent risk factors were the same for atopic asthma as for all asthma for Black Caribbeans, Black Africans and Bangladeshis (Additional file 1: Table S3). There was some small variation in risk factors for the other groups. For White UK, gender was an additional correlate, with girls more likely to report atopic asthma than boys. Among Indians, unlike the reporting of all asthma, girls were no more likely than boys to report atopic asthma and grandparental asthma was an additional correlate. TDS was a correlate of atopic asthma in Pakistanis. Among Mixed White/Black Caribbeans, parental asthma was the only significant correlate. There was a significant interaction between BMI and gender for Black Africans. In models stratified by gender, the association between BMI and asthma was greater for boys (Odds ratio for boys 1.55, 95%CI: 1.13-2.13).

Children who were born abroad and resident in the UK ≤ 5 yrs were less likely to report at least one parent who had asthma than those born in the UK (UK born 13%, 95%CI: 12-14%, ≤ 5 yrs residence 7%, 5-9%). Additional file 1: Table S4 shows, however, that parental asthma was a significant influence on own asthma regardless of generational status, and that for atopic asthma the effect was greater in those who resided in the UK ≤ 5 yrs than those born in the UK. This effect was unchanged when adjusted for ethnicity, and there were no significant interactions between ethnicity and parental asthma in the models.

The effect of controlling for potential risk factors on ethnic differences in all asthma and atopic asthma is shown in Additional file 1: Tables S5 and S6. These models were stratified by gender due to the interaction with ethnicity. Additional file 1: Table S5 shows that on adjustment for familial asthma, Black Caribbean boys were more likely to report asthma than White UK boys. Adjustment for generational status removed the asthma advantage in Black African boys and girls, and Bangladeshi girls. Adjustment for the better psychological well-being of Indian girls reduced their asthma advantage. Excess asthma in the Mixed White/Black Caribbean boys remained after these adjustments. The corresponding results are shown for atopic asthma in Additional file 1: Table S6. Atopic asthma advantage in Black African girls was associated with being born in Africa and with less of a family history of asthma. In Indian and Bangladeshi girls it was associated with their better psychological well-being

## Discussion

This is the first known UK study to systematically examine the effect of generational status, family history of asthma, psychological well-being and body size on asthma prevalence in ethnic minority young adolescents. Poor psychological well-being and a family history of asthma were associated with asthma in every ethnic group. Generational status played a role in the asthma advantage of some ethnic minority groups, recent migration to the UK associated with a significant asthma advantage.

A review of 33 studies from various countries reported odds ratios of between two and four for the effect of one or more first degree relatives having asthma on childhood asthma[15]. This is comparable with the effect size reported in each ethnic group here. In the UK the National Study of Health and Growth (NSHG) investigated the association of familial asthma with ethnic differences in asthma, wheeze and atopy in children aged 5 to 11 yrs[28]. The effect size reported for family history of asthma or wheeze (odds ratio 2.42 associated with maternal asthma, 2.41 with paternal asthma) were similar to those we have reported. Few studies of familial asthma have been reported from home countries of children in DASH. Having a parent with asthma has been associated with a 2-4 fold increase in asthma in 8-17 year olds in Ghana[29], in 14-17 yr olds in India[30], and in 1-10 yr olds in Sri Lanka[31]. In DASH, ethnic differences in inadequate description of family asthma may have had some effect on the effect sizes associated with family asthma. Black African origin children were generally more likely to give an inadequate description of parental or grandparental asthma status. This may be due to ethnic specific differences in the nature of migration, Black Africans more likely to have been born abroad and less likely to live with grandparents. Indians were least likely to inadequately report parental asthma status and almost all in this group lived with two parents compared with about half of the Black Caribbeans. Other than for Pakistanis (OR 4.69), it is reassuring that the ethnic specific effect sizes for parental asthma in DASH are comparable to those reported in other studies[15,28-31].

A previous UK study of South Asian (Indian, Pakistani or Bangladeshi) women found that those who were born abroad and had migrated to the UK aged 5 yrs or older had significantly reduced risk of asthma than those born in the UK or those who had migrated at a younger age[19]. This partially corresponds with our results as adjustment for generation status removed the asthma advantage for Bangladeshi girls, although not for Indian or Pakistani girls. Length of exposure to the environments of developed countries has been shown to have an adverse effect on asthma risk in children in two other studies[3,20]. Mexican children born in Mexico have been found to have lower rates of asthma and wheeze than those born in the US[20]. Subramanian et al recently reported supporting findings for the effect of nativity. In a US sample of white, Hispanic and Black US children, immigrant mothers and the children of immigrant mothers had a lower risk of asthma than those born in the US regardless of ethnicity[32]. In Australia, the frequency of wheeze among teenagers from a range of countries (including Africa and South Asia) increased with length of residence in Australia[3].

Given that those born abroad were less likely to report familial asthma than those born in the UK, it is possible that duration of residence captures not only the effect of adverse exposures in the UK environment but also susceptibility to asthma. It is interesting to note that less than 6 years of residence in the UK had an independent protective effect here for Black Caribbeans and Black Africans, possibly reflecting continuing protection from early life exposures in home countries. The study of South Asian women suggested a critical period up to 5 years of age in home countries as being protective[19]. In DASH it is not possible to analyse age at migration with accuracy; pupils resident in the UK for ≤ 5 yrs were aged between 6 and 13 years on arrival to the UK. Central heating and use of chemical based cleaning products in UK homes have been found to affect wheeze in childhood[33,34]. The rising prevalence of childhood asthma symptoms in developing countries (including Africa, the Caribbean and South Asia)[35] suggest that the relative protection for children abroad observed in our study may change.

In DASH, SES appeared to only have an effect in Black Africans (with less asthma in the least advantaged) and did not explain the higher reporting in Black Caribbean or Mixed White/Black Caribbean boys. This is in contrast to findings from a previous UK study of 3 years olds[7] and also of some US studies that have shown that a higher prevalence of asthma in Black than White Americans is due to more economic disadvantage[36]. Some studies have reported a negative association[37,38] (as we have found here with Africans), and between country variation in asthma suggests higher prevalence in affluent countries[39]. We cannot discount that our results may be related to the measure of SES we used. Measuring self reported socio-economic status among children is problematic. Furthermore, other work has shown that multidimensional indices of socio-economic status maybe more relevant as a measure of inequality among ethnic minorities, given the disruption of occupational careers and general life changes on migration[23,24]. The distribution of the standard of living measure by ethnicity in DASH was as expected from national surveys, with all groups except Indians significantly less likely to be in the most advantaged top tertile than White UK. Reported paternal economic activity and family type provided some verification of our measure of socio-economic status as children with a working parent and those in two-parent households were most likely to be in the most advantaged top tertile (32%) and least likely to be in the least advantaged tertile (21%) of the standard of living measure. Neither parental economic activity nor family structure was consistently related to asthma.

Psychosocial stress has previously been associated with the initiation and exacerbation of asthma and wheeze in children and adolescents[40] but has not been examined in relation to ethnicity. Chronic stress has also been associated with increased production of Interleukin 5 and 15, and eosinophil counts in the inflammatory pathway in asthmatics[41], and this association may explain the reported pathway between SES and inflammation processes. Better psychological well-being has been reported for ethnic minorities in DASH, particularly for Black Africans, and this was not associated with SES[42]. We found a consistent association of psychological well-being with asthma in every group, after adjustment for SES. It has been suggested that the stress of migration due to dissociation from family and neighbourhood support networks may increase asthma risk[18]. This may be the case and as with all cross-sectional studies we cannot rule out reverse causality (in that asthma influences psychological well-being).

Our study is subject to limitations. It has been suggested that ethnic minority groups, particularly South Asians, may under-report asthma. In DASH, children self-identified as asthmatic or as having asthma symptoms (wheeze and/or breathing difficulties). There were no ethnic differences in report of wheeze and/or breathing difficulties (data not shown). It is possible that if prevalence of asthma in family is low this may lead to less awareness of asthma and its symptoms (i.e. wheeze) in children so we can not rule out some under-reporting. We can not rule out the possibility of underreporting by some ethnic minority groups due to reduced access to health care services and hence opportunity for asthma diagnosis. Children from other Mixed ethnicity groups were excluded from the main analysis as they were from a heterogeneous group of numerous ethnicities with too small a sample from each specific group (other than the Mixed Black Caribbean/White UK group). Asthma prevalence in this other Mixed ethnicity group was 34% (95% CI 28, 40%, not statistically different to the White UK group). It is conceptually confusing to combine these groups with different cultural, biological and material exposures, and it is difficult to interpret the results. Mixed ethnicity is the future of Britain but the meaning of this category needs further research. The nature of school-based studies may mean that school avoiders, in particular long-term truants, and pupils who were more likely to be absent due to illness were under represented in the sample. This group may be more likely to live in poor social conditions and have poor psychological well-being. The cross sectional nature of this study prevents conclusions on a causal relationship between asthma with obesity or psychological well-being in ethnic minorities. The recent follow up of DASH adolescents aged 14-16 yrs will provide more detailed data on growth and asthma.

## Conclusion

These findings highlight the ethnic variation in asthma risk, with Black Caribbean and Mixed Black Caribbean/White boys having a higher risk than White UK, but other minorities having a similar or lower risk. Family history of asthma and psychological well-being were consistent correlates for asthma regardless of ethnicity. Duration of residence for less than 5 years in the UK had a protective effect for Black Caribbeans and Black Africans. Body size was a correlate of asthma in Black Africans, boys in particular. It is important not to be complacent towards the lower risk in some groups. Comparative research in developed and developing countries would greatly aid the understanding of early protective influences on asthma risk.

## Competing interests

The authors declare that they have no competing interests.

## Authors' contributions

MW produced the first draft of the manuscript and conducted the analysis. MW and SH formulated the research question, redrafted the paper, are the guarantors of the paper and read and approved the final version. SH is the Principal investigator of DASH.

## Pre-publication history

The pre-publication history for this paper can be accessed here:

http://www.biomedcentral.com/1471-2431/10/18/prepub

## Supplementary Material

Additional file 1 **Supplemental tables**. Supplemental tables S1 - S6. Click here for file
