# Supplementary material for: Asthma in Black African, Black Caribbean and South Asian adolescents in the MRC DASH study: a cross sectional analysis
Source: BMC Pediatr. 2010 Mar 25;10:18. doi: 10.1186/1471-2431-10-18 (PMC2851680; doi:10.1186/1471-2431-10-18)
Supplement: Additional file 1 — Supplemental tables. Supplemental tables S1 - S6. [file 1471-2431-10-18-S1.DOC]

Table 1 Reported prevalence of asthma and family history of asthma, and social and psychosocial characteristics by ethnicity - % (95%CI)

|  | White UK | Black Caribbean | Black African | Indian | Pakistani | Bangladeshi | Mixed, White/ Black Caribbean |
| --- | --- | --- | --- | --- | --- | --- | --- |
| N (%) | 1219 (19) | 933 (14) | 1095 (17) | 459 (7) | 215 (3) | 392 (6) | 299 (5) |
| *Asthma* |  |  |  |  |  |  |  |
| Asthma^‡^ | 32 (30, 35) | 33 (30, 36) | 23 (20, 25)**^†^** | 24 (20, 28)**^†^** | 29 (24, 36) | 26 (22, 30)**^†^** | 40 (35, 46)* |
| Atopic asthma^‡^ | 16 (14, 18) | 20 (17, 22)* | 11 (9, 13)**^†^** | 12 (9, 15)**^†^** | 12 (8, 17) | 12 (9, 16)**^†^** | 24 (19 ,29)* |
| Hay fever | 25 (22, 27) | 33 (30, 36)* | 24 (22, 27) | 20 (17, 24) | 25 (20, 31) | 20 (16, 24) | 34 (29, 40)* |
| Eczema | 18 (16, 20) | 18 (16, 21) | 13 (11, 15)**^†^** | 13 (10, 16)**^†^** | 10 (7, 15) | 12 (9, 16) | 18 (14, 23) |
| *Familial asthma* |  |  |  |  |  |  |  |
| ≥1 parent asthma | 17 (15, 19) | 9 (8, 11)**^†^** | 6 (5, 7)**^†^** | 16 (13, 19)**^†^** | 16 (12, 21) | 14 (11, 18)**^†^** | 16 (12, 20) |
| *Inadequately described***^§^** | *30 (28, 33)* | *53 (50, 56)** | *43 (40, 46)** | *14 (11, 17)***^†^** | *23 (18, 29)***^†^** | *21 (17, 26)***^†^** | *47 (42, 53)** |
| ≥1 grandparent asthma | 12 (11, 14) | 7 (6, 9)**^†^** | 4 (3, 5)**^†^** | 10 (8, 13) | 7 (5, 12) | 10 (7, 13) | 10 (7, 14) |
| *Inadequately described***^§^** | *29 (26, 31)* | *32 (29, 35)* | *44 (41, 47)** | *30 (26, 34)* | *40 (33, 46)** | *30 (26, 35)* | *35 (30, 40)* |
| *Generational status* |  |  |  |  |  |  |  |
| UK born | 97 (95, 97) | 72 (69, 75)**^†^** | 55 (52, 58)**^†^** | 79 (75, 82)**^†^** | 81 (75, 86)**^†^** | 77 (72, 81)**^†^** | 93 (90, 96) |
| Born abroad, resident in UK >5yrs | 1 (1, 2) | 10 (8, 12)* | 19 (17, 22)* | 6 (4, 9)* | 10 (6, 15)* | 11 (8, 14)* | 2 (1, 4) |
| Born abroad, resident in UK ≤5yrs | 0 (0, 0) | 14 (12, 17)* | 19 (17, 22)* | 14 (11, 17)* | 6 (3, 10)* | 9 (7, 12)* | 2 (1, 5)* |
| ≥1 parent born abroad | 13 (12, 15) | 75 (72, 78)* | 95 (94, 97)* | 95 (93, 97)* | 98 (95, 99)* | 94 (91, 96)* | 46 (41, 52)* |
| 4 grandparents born abroad | 4 (3, 6) | 67 (64, 70)* | 74 (71, 77)* | 63 (59, 67)* | 74 (68, 80)* | 71 (66, 75)* | 15 (11, 19)* |
| *Smoking* |  |  |  |  |  |  |  |
| ≥1 parent smoking | 50 (47, 53) | 34 (31, 37)**^†^** | 12 (10, 14)**^†^** | 22 (18, 26)**^†^** | 41 (35, 48)**^†^** | 27 (23, 32)**^†^** | 56 (50, 61) |
| *SES°* |  |  |  |  |  |  |  |
| Most advantaged 1^st^ tertile | 39 (37, 42) | 26 (24, 29) | 18 (16, 20) | 37 (32, 41) | 16 (12, 22) | 29 (24, 33) | 29 (24, 35) |
| Least advantaged 3^rd^ tertile | 20 (18, 23) | 25 (22, 28) | 30 (28, 33)* | 18 (14, 21) | 32 (26, 39)* | 23 (20, 28) | 23 (19, 29) |
| *Psychological well-being*– *mean TDS* ^§^ *(SE)* | 12 (0.2) | 11 (0.2) | 11 (0.2)**^†^** | 10 (0.2)**^†^** | 11 (0.4) | 11 (0.3)**^†^** | 11 (0.3) |
| *Body size- mean,* BMI percentile *(SE)* | 63 (0.9) | 69 (1.0)* | 69 (0.9)* | 57 (1.7)**^†^** | 58 (2.2)**^†^** | 61 (1.7) | 67 (1.7) |

* Significantly (P<0.05) higher than White UK

**^†^** Significantly (P<0.05) lower than White UK

^‡^ asthma - asthma or wheeze or breathing difficulties, atopic – with hay fever, eczema or skin allergies, non-atopic – without

**^§^** Inadequately described – child did not answer the question, writing was unintelligible or written “don’t know”

Table 2 Asthma^†^: risk factors within ethnic groups

|  | White UK | Black Caribbean | Black African | Indian | Pakistani | Bangladeshi | Mixed – White/ Black Caribbean |
| --- | --- | --- | --- | --- | --- | --- | --- |
|  | Odds Ratio (95%CI)^ll^ | Odds Ratio (95%CI)^ll^ | Odds Ratio (95%CI)^ll^ | Odds Ratio (95%CI)^ll^ | Odds Ratio (95%CI)^ll^ | Odds Ratio (95%CI)^ll^ | Odds Ratio (95%CI)^ll^ |
| Male=1.00 |  |  |  |  |  |  |  |
| Female | 1.10 (0.85 - 1.43) | 0.74 (0.54 - 1.03) | 1.01 (0.73 - 1.41) | 0.56 (0.33 - 0.94)* | 0.94 (0.44 - 2.01) | 0.58 (0.33 - 1.04) | 0.83 (0.49 - 1.42) |
|  |  |  |  |  |  |  |  |
| Pupil UK born=1.00 |  |  |  |  |  |  |  |
| Born abroad, UK resident >5yrs | 0.17 (0.02 - 1.38) | 0.72 (0.43 - 1.21) | 0.84 (0.55 - 1.29) | 0.97 (0.33 - 2.84) | 0.81 (0.23 - 2.87) | 0.72 (0.29 - 1.83) | - |
| Born abroad, UK resident ≤5yrs | - | 0.48 (0.29 - 0.80)* | 0.63 (0.40 - 1.00)* | 0.75 (0.33 - 1.69) | - | 0.72 (0.27 - 1.93) | 0.79 (0.11 - 5.49) |
| Inadequately described*^§^* | 0.71 (0.25 - 2.01) | 0.72 (0.30 - 1.71) | 0.87 (0.41 - 1.85) | 1.16 (0.17 - 7.90) | 0.59 (0.07 - 5.09) | 0.80 (0.11 - 5.66) | 1.31 (0.24 - 7.27) |
|  |  |  |  |  |  |  |  |
| No parents have asthma=1.00 |  |  |  |  |  |  |  |
| ≥1 parent with asthma | 1.77 (1.23 - 2.56)* | 2.59 (1.49 - 4.48)* | 1.99 (1.07 - 3.69)* | 2.66 (1.41 - 5.01)* | 4.69 (1.72 - 12.81)* | 3.07 (1.49 - 6.31)* | 1.71 (0.72 - 4.04) |
| Inadequately described*^§^* | 0.97 (0.63 - 1.48) | 0.81 (0.49 - 1.35) | 1.17 (0.67 - 2.04) | 1.26 (0.52 - 3.06) | 1.05 (0.32 - 3.44) | 1.97 (0.78 - 4.93) | 1.14 (0.42 - 3.07) |
|  |  |  |  |  |  |  |  |
| No grandparents have asthma=1.00 |  |  |  |  |  |  |  |
| ≥1 grandparent with asthma | 2.35 (1.60 - 3.45)* | 2.80 (1.59 - 4.96)* | 2.65 (1.30 - 5.42)* | 2.14 (1.00 - 4.60) | 0.74 (0.18 - 3.02) | 2.23 (0.96 - 5.21) | 1.91 (0.79 - 4.63) |
| Inadequately described*^§^* | 1.35 (0.99 - 1.83) | 1.20 (0.85 - 1.70) | 1.27 (0.92 - 1.76) | 1.43 (0.81 - 2.51) | 1.98 (0.91 - 4.30) | 1.55 (0.85 - 2.83) | 1.08 (0.59 - 1.98) |
|  |  |  |  |  |  |  |  |
| No parents smoke at home=1.00 |  |  |  |  |  |  |  |
| ≥1 parent smokes | 1.26 (0.96 - 1.67) | 1.43 (1.02 - 2.01)* | 1.07 (0.66 - 1.74) | 0.66 (0.35 - 1.24) | 1.27 (0.59 - 2.70) | 0.91 (0.50 - 1.67) | 1.24 (0.68 - 2.24) |
| Inadequately described*^§^* | 1.27 (0.72 - 2.25) | 1.15 (0.70 - 1.88) | 0.76 (0.41 - 1.40) | 0.46 (0.11 - 1.89) | 1.75 (0.40 - 7.72) | 0.77 (0.22 - 2.67) | 1.63 (0.54 - 4.99) |
|  |  |  |  |  |  |  |  |
| SES, most advantaged tertile=1.00 |  |  |  |  |  |  |  |
| Mid advantaged tertile | 0.85 (0.62 - 1.16) | 0.78 (0.53 - 1.15) | 0.87 (0.58 - 1.33) | 0.72 (0.40 - 1.29) | 0.69 (0.24 - 1.99) | 0.72 (0.38 - 1.39) | 0.82 (0.41 - 1.63) |
| Least advantaged tertile | 1.06 (0.73 - 1.53) | 0.96 (0.62 - 1.50) | 0.57 (0.36 - 0.91)* | 0.61 (0.28 - 1.35) | 0.62 (0.20 - 1.89) | 0.72 (0.34 - 1.54) | 1.02 (0.47 - 2.19) |
| Inadequately described*^§^* | 1.14 (0.66 - 1.96) | 0.88 (0.53 - 1.47) | 0.67 (0.38 - 1.17) | 1.79 (0.75 - 4.26) | 0.38 (0.08 - 1.73) | 2.55 (0.98 - 6.66) | 0.95 (0.35 - 2.62) |
|  |  |  |  |  |  |  |  |
| Psychological well-being (TDS) *^‡^* | 1.04 (1.02 - 1.07)* | 1.06 (1.03 - 1.09)* | 1.07 (1.04 - 1.10)* | 1.07 (1.02 - 1.12)* | 1.07 (1.00 - 1.14) | 1.09 (1.04 - 1.15)* | 1.06 (1.00 - 1.11)* |
|  |  |  |  |  |  |  |  |
| Body size (BMI percentile) | 1.04 (0.94 - 1.16) | 0.94 (0.83 - 1.05) | 1.15 (1.01 - 1.31)* | 1.07 (0.91 - 1.26) | 1.23 (0.93 - 1.62) | 0.95 (0.78 - 1.15) | 1.08 (0.86 - 1.34) |

*^*^ P<0.05 compared to reference group, ^†^ Asthma or wheeze or breathing difficulties, ^‡^ Total difficulties score from Goodman’s Strength and Difficulties Questionnaire*

*^§^ Inadequately described – child did not answer the question, writing was unintelligible or written “don’t know”, ^ll^ Adjusted for all variables in the table, and age, family type, mother and father employment status, number of siblings, parental and grandparental generational status.* *Clustering in schools was accounted for using the xtlogit command in Stata with the random effect option*

Table 3 Atopic asthma^†^: risk factors within ethnic groups

|  | White UK | Black Caribbean | Black African | Indian | Pakistani | Bangladeshi | Mixed – White/ Black Caribbean |
| --- | --- | --- | --- | --- | --- | --- | --- |
|  | Odds Ratio (95%CI)^ll^ | Odds Ratio (95%CI)^ll^ | Odds Ratio (95%CI)^ll^ | Odds Ratio (95%CI)^ll^ | Odds Ratio (95%CI)^ll^ | Odds Ratio (95%CI)^ll^ | Odds Ratio (95%CI)^ll^ |
| Male=1.00 |  |  |  |  |  |  |  |
| Female | 1.61 (1.13 - 2.28)* | 0.88 (0.60 - 1.28) | 1.03 (0.67 - 1.58) | 0.69 (0.35 - 1.34) | 0.73 (0.21 - 2.57) | 0.49 (0.21 - 1.12) | 0.84 (0.40 - 1.77) |
|  |  |  |  |  |  |  |  |
| Pupil UK born=1.00 |  |  |  |  |  |  |  |
| Born abroad, UK resident >5yrs | 0.47 (0.06 - 3.91) | 0.53 (0.27 - 1.04) | 0.64 (0.35 - 1.17) | 1.15 (0.33 - 3.99) | 0.63 (0.04 - 10.44) | 0.61 (0.15 - 2.47) | - |
| Born abroad, UK resident ≤5yrs | - | 0.21 (0.09 - 0.46)* | 0.42 (0.21 - 0.83)* | 0.54 (0.17 - 1.69) | - | 0.49 (0.08 - 2.95) | - |
| Inadequately described*^§^* | 0.65 (0.13 - 3.36) | 0.36 (0.10 - 1.35) | 0.87 (0.29 - 2.63) | 1.03 (0.06 - 16.55) | - | - | 0.84 (0.08 - 8.65) |
|  |  |  |  |  |  |  |  |
| No parents have asthma=1.00 |  |  |  |  |  |  |  |
| ≥1 parent with asthma | 2.22 (1.39 - 3.53)* | 2.28 (1.21 - 4.29)* | 2.26 (1.01 - 5.08)* | 2.31 (1.07 - 4.99)* | 5.47 (1.20 - 24.96)* | 4.61 (1.83 - 11.63)* | 3.55 (1.11 - 11.40)* |
| Inadequately described*^§^* | 1.04 (0.60 - 1.83) | 0.60 (0.32 - 1.13) | 1.36 (0.64 - 2.88) | 0.32 (0.06 - 1.66) | 1.02 (0.14 - 7.22) | 2.85 (0.85 - 9.58) | 1.43 (0.39 - 5.25) |
|  |  |  |  |  |  |  |  |
| No grandparents have asthma=1.00 |  |  |  |  |  |  |  |
| ≥1 grandparent with asthma | 2.36 (1.45 - 3.83)* | 2.35 (1.18 - 4.68)* | 2.85 (1.08 - 7.53)* | 3.14 (1.24 - 7.97)* | 0.72 (0.08 - 6.57) | 1.88 (0.56 - 6.32) | 2.71 (0.87 - 8.49) |
| Inadequately described*^§^* | 1.30 (0.87 - 1.96) | 1.51 (1.00 - 2.29)* | 1.63 (1.05 - 2.52)* | 2.15 (1.01 - 4.61)* | 2.28 (0.60 - 8.68) | 1.29 (0.56 - 2.95) | 1.07 (0.47 - 2.45) |
|  |  |  |  |  |  |  |  |
| No parents smoke at home=1.00 |  |  |  |  |  |  |  |
| ≥1 parent smokes | 1.39 (0.96 - 2.01) | 1.60 (1.06 - 2.40)* | 1.35 (0.74 - 2.46) | 0.66 (0.28 - 1.55) | 0.83 (0.23 - 2.94) | 1.29 (0.59 - 2.83) | 1.93 (0.83 - 4.48) |
| Inadequately described*^§^* | 1.10 (0.48 - 2.52) | 1.48 (0.82 - 2.68) | 0.39 (0.14 - 1.05) | 1.09 (0.17 - 6.98) | 0.53 (0.03 - 9.81) | 0.30 (0.02 - 3.65) | 5.15 (1.30 - 20.31) |
|  |  |  |  |  |  |  |  |
| SES, most advantaged tertile=1.00 |  |  |  |  |  |  |  |
| Mid advantaged tertile | 0.83 (0.54 - 1.25) | 0.66 (0.42 - 1.04) | 0.90 (0.52 - 1.57) | 1.00 (0.46 - 2.16) | 0.38 (0.07 - 2.22) | 1.05 (0.43 - 2.58) | 0.84 (0.32 - 2.20) |
| Least advantaged tertile | 1.08 (0.66 - 1.76) | 0.79 (0.46 - 1.34) | 0.52 (0.28 - 0.97)* | 1.12 (0.39 - 3.19) | 0.12 (0.02 - 0.87)* | 1.01 (0.36 - 2.82) | 1.33 (0.44 - 4.03) |
| Inadequately described*^§^* | 0.38 (0.14 - 0.99) | 0.73 (0.39 - 1.39) | 0.58 (0.27 - 1.28) | 2.33 (0.77 - 6.99) | 0.09 (0.01 - 1.39) | 0.85 (0.14 - 5.05) | 2.30 (0.57 - 9.38) |
|  |  |  |  |  |  |  |  |
| Psychological well-being (TDS)*^‡^* | 1.06 (1.03 - 1.09)* | 1.06 (1.02 - 1.10)* | 1.06 (1.02 - 1.11)* | 1.09 (1.02 - 1.16)* | 1.22 (1.06 - 1.40)* | 1.13 (1.05 - 1.21)* | 1.03 (0.95 - 1.10) |
|  |  |  |  |  |  |  |  |
| Body size (BMI percentile) | 1.04 (0.90 - 1.20) | 0.89 (0.77 - 1.03) | 1.18 (0.99 - 1.41) | 1.05 (0.85 - 1.30) | 1.23 (0.79 - 1.92) | 0.80 (0.62 - 1.05) | 1.09 (0.81 - 1.48) |

*^*^ P<0.05 compared to reference group, ^†^ Asthma or wheeze or breathing difficulties, ^‡^ Total difficulties score from Goodman’s Strength and Difficulties Questionnaire*

*^§^ Inadequately described – child did not answer the question, writing was unintelligible or written “don’t know”, ^ll^ Adjusted for all variables in the table, and age, family type, mother and father employment status, number of siblings, parental and grandparental generational status.* *Clustering in schools was accounted for using the xtlogit command in Stata with the random effect option*

Table 4 The association between family history of asthma and childhood asthma^†^, stratified by generational status

|  | UK born | Born Abroad, resident >5yrs | Born Abroad, resident ≤5yrs |
| --- | --- | --- | --- |
|  | Odds Ratio (95%CI)^§^ | Odds Ratio (95%CI)^§^ | Odds Ratio (95%CI)^§^ |
| All asthma | n=4637 | n=760 | n=770 |
| No parents have asthma=1.00 |  |  |  |
| ≥1 parent with asthma | 2.33 (1.91 - 2.83)* | 1.58 (0.88 - 2.84) | 4.84 (2.42 - 9.67)* |
| Inadequately described^‡^ | 1.17 (0.94 - 1.46) | 0.75 (0.41 - 1.40) | 0.73 (0.37 - 1.44) |
|  |  |  |  |
| Atopic asthma | n=3973 | n=654 | n=673 |
| No parents have asthma=1.00 |  |  |  |
| ≥1 parent with asthma | 2.44 (1.91 - 3.10)* | 1.88 (0.84 - 4.21) | 9.28 (3.39 - 25.39)* |
| Inadequately described^‡^ | 1.03 (0.78 - 1.37) | 0.58 (0.23 - 1.50) | 0.49 (0.13 - 1.80) |

*^*^ P<0.05 compared to reference group*

^†^ *Atopic – asthma with hay fever or eczema, model excludes non-atopic asthmatics*

*^‡^ Inadequately described – child did not answer the question, writing was unintelligible or written “don’t know”*

^§^ *Adjusted for age, sex, SES, family type, mother and father employment status, number of siblings, parental smoking and TDS. Clustering in schools was accounted for using the xtlogit command in Stata with the random effect option*

Table 5 Asthma†: ethnic differences adjusted for potential risk factors

|  | Black Caribbean | Black African | Indian | Pakistani | Bangladeshi | Mixed – White/ Black Caribbean |
| --- | --- | --- | --- | --- | --- | --- |
|  | Odds Ratio (95%CI)^§^ | Odds Ratio (95%CI)^§^ | Odds Ratio (95%CI)^§^ | Odds Ratio (95%CI)^§^ | Odds Ratio (95%CI)^§^ | Odds Ratio (95%CI)^§^ |
| *Boys (n=3411)* |  |  |  |  |  |  |
| Model 1 | 1.24 (0.96 - 1.62) | 0.65 (0.49 - 0.86)* | 0.96 (0.68 - 1.34) | 1.04 (0.68 - 1.58) | 0.96 (0.69 - 1.34) | 1.65 (1.13 - 2.41)* |
| Model 2 | 1.19 (0.92 - 1.56) | 0.63 (0.47 - 0.85)* | 0.97 (0.69 - 1.36) | 1.08 (0.69 - 1.67) | 0.99 (0.70 - 1.40) | 1.62 (1.10 - 2.36)* |
| Model 3 | 1.33 (0.94 - 1.88) | 0.74 (0.51 - 1.07) | 1.02 (0.68 - 1.54) | 1.11 (0.68 - 1.82) | 1.05 (0.69 - 1.59) | 1.64 (1.08 - 2.50)* |
| Model 4 | 1.46 (1.02 - 2.07)* | 0.81 (0.55 - 1.18) | 1.00 (0.66 - 1.52) | 1.13 (0.68 - 1.87) | 1.02 (0.66 - 1.56) | 1.60 (1.05 - 2.46)* |
| Model 5 | 1.48 (1.04 - 2.10)* | 0.84 (0.57 - 1.23) | 1.03 (0.68 - 1.57) | 1.14 (0.69 - 1.89) | 1.04 (0.68 - 1.60) | 1.58 (1.03 - 2.42)* |
| Model 6 | 1.48 (1.03 - 2.11)* | 0.90 (0.61 - 1.33) | 1.06 (0.69 - 1.62) | 1.16 (0.70 - 1.94) | 1.11 (0.72 - 1.71) | 1.65 (1.07 - 2.56)* |
| Model 7 | 1.49 (1.04 - 2.13)* | 0.91 (0.62 - 1.34) | 1.08 (0.71 - 1.65) | 1.18 (0.71 - 1.97) | 1.14 (0.74 - 1.75) | 1.66 (1.07 - 2.56)* |
| *Girls (n=3053)* |  |  |  |  |  |  |
| Model 1 | 0.87 (0.66 - 1.14) | 0.56 (0.43 - 0.73)* | 0.41 (0.27 - 0.62)* | 0.75 (0.44 - 1.26) | 0.45 (0.28 - 0.72)* | 1.16 (0.80 - 1.69) |
| Model 2 | 0.84 (0.63 - 1.11) | 0.56 (0.43 - 0.75)* | 0.44 (0.29 - 0.66)* | 0.85 (0.49 - 1.46) | 0.48 (0.30 - 0.79)* | 1.09 (0.74 - 1.59) |
| Model 3 | 1.05 (0.73 - 1.52) | 0.75 (0.51 - 1.10) | 0.56 (0.35 - 0.91)* | 1.00 (0.55 - 1.83) | 0.60 (0.35 - 1.03) | 1.20 (0.79 - 1.84) |
| Model 4 | 1.08 (0.74 - 1.58) | 0.82 (0.56 - 1.22) | 0.56 (0.34 - 0.91)* | 1.00 (0.54 - 1.86) | 0.66 (0.38 - 1.16) | 1.27 (0.83 - 1.96) |
| Model 5 | 1.07 (0.73 - 1.56) | 0.87 (0.59 - 1.30) | 0.59 (0.36 - 0.96)* | 1.00 (0.54 - 1.87) | 0.68 (0.39 - 1.19) | 1.25 (0.81 - 1.93) |
| Model 6 | 1.09 (0.74 - 1.60) | 0.91 (0.61 - 1.36) | 0.65 (0.40 - 1.08) | 1.04 (0.55 - 1.94) | 0.72 (0.41 - 1.26) | 1.31 (0.85 - 2.03) |
| Model 7 | 1.06 (0.72 - 1.56) | 0.88 (0.59 - 1.32) | 0.67 (0.40 - 1.10) | 1.06 (0.56 - 1.98) | 0.74 (0.42 - 1.29) | 1.30 (0.84 - 2.01) |

*^*^ P<0.05 compared to reference group, ^†^ Asthma or wheeze or breathing difficulties,*  ^§^ *White UK = baseline,* *clustering in schools was accounted for using the xtlogit command in Stata with the random effect option*

Model 1: Adjusted for age

Model 2: Adjusted for (1) and disadvantage, maternal employment status, paternal employment status, family type, number of siblings

Model 3: Adjusted for (2) and generational status (pupil, parent and grandparent born abroad status)

Model 4: Adjusted for (3) and family history of asthma (parental and grandparental)

Model 5: Adjusted for (4) and parental smoking

Model 6: Adjusted for (5) and psychological well-being

Model 7: Adjusted for (6) and BMI percentile

Table 6 Atopic asthma^†^: ethnic differences adjusted for potential risk factors

|  | Black Caribbean | Black African | Indian | Pakistani | Bangladeshi | Mixed – White/ Black Caribbean |
| --- | --- | --- | --- | --- | --- | --- |
|  | Odds Ratio (95%CI)^§^ | Odds Ratio (95%CI)^§^ | Odds Ratio (95%CI)^§^ | Odds Ratio (95%CI)^§^ | Odds Ratio (95%CI)^§^ | Odds Ratio (95%CI)^§^ |
| *Boys (n=3411)* |  |  |  |  |  |  |
| Model 1 | 1.76 (1.24 - 2.48)* | 0.83 (0.56 - 1.21) | 1.07 (0.68 - 1.70) | 1.12 (0.62 - 2.01) | 1.19 (0.76 - 1.86) | 2.55 (1.60 - 4.06)* |
| Model 2 | 1.75 (1.23 - 2.49)* | 0.88 (0.59 - 1.31) | 1.15 (0.72 - 1.84) | 1.36 (0.74 - 2.50) | 1.44 (0.90 - 2.29) | 2.56 (1.60 - 4.10)* |
| Model 3 | 2.20 (1.38 - 3.50)* | 1.22 (0.73 - 2.03) | 1.32 (0.75 - 2.32) | 1.53 (0.77 - 3.05) | 1.68 (0.95 - 2.95) | 2.70 (1.58 - 4.62)* |
| Model 4 | 2.49 (1.54 - 4.01)* | 1.39 (0.82 - 2.34) | 1.34 (0.75 - 2.38) | 1.59 (0.79 - 3.22) | 1.68 (0.94 - 3.00) | 2.58 (1.49 - 4.46)* |
| Model 5 | 2.56 (1.59 - 4.14)* | 1.52 (0.89 - 2.57) | 1.43 (0.80 - 2.55) | 1.65 (0.82 - 3.34) | 1.77 (0.99 - 3.18) | 2.46 (1.42 - 4.26)* |
| Model 6 | 2.50 (1.54 - 4.07)* | 1.55 (0.91 - 2.65) | 1.43 (0.80 - 2.58) | 1.57 (0.76 - 3.23) | 1.80 (1.00 - 3.24) | 2.51 (1.44 - 4.39)* |
| Model 7 | 2.54 (1.56 - 4.13)* | 1.58 (0.92 - 2.70) | 1.47 (0.82 - 2.65) | 1.60 (0.77 - 3.29) | 1.86 (1.03 - 3.36)* | 2.53 (1.45 - 4.43)* |
| *Girls (n=3053)* |  |  |  |  |  |  |
| Model 1 | 0.95 (0.69 - 1.31) | 0.47 (0.33 - 0.66)* | 0.40 (0.24 - 0.67)* | 0.51 (0.24 - 1.07) | 0.31 (0.16 - 0.61)* | 1.16 (0.74 - 1.83) |
| Model 2 | 0.93 (0.67 - 1.30) | 0.49 (0.35 - 0.71)* | 0.42 (0.25 - 0.71)* | 0.66 (0.31 - 1.42) | 0.35 (0.18 - 0.70)* | 1.10 (0.69 - 1.73) |
| Model 3 | 1.02 (0.66 - 1.58) | 0.62 (0.38 - 0.99)* | 0.49 (0.27 - 0.89)* | 0.67 (0.29 - 1.53) | 0.39 (0.18 - 0.82)* | 1.05 (0.63 - 1.75) |
| Model 4 | 1.07 (0.68 - 1.69) | 0.67 (0.41 - 1.10) | 0.47 (0.26 - 0.87)* | 0.67 (0.28 - 1.57) | 0.42 (0.20 - 0.91)* | 1.11 (0.65 - 1.87) |
| Model 5 | 1.07 (0.68 - 1.69) | 0.75 (0.46 - 1.24) | 0.51 (0.28 - 0.95)* | 0.68 (0.29 - 1.60) | 0.45 (0.21 - 0.97)* | 1.09 (0.64 - 1.85) |
| Model 6 | 1.10 (0.69 - 1.76) | 0.82 (0.49 - 1.36) | 0.59 (0.32 - 1.10) | 0.72 (0.30 - 1.72) | 0.50 (0.23 - 1.08) | 1.16 (0.68 - 1.99) |
| Model 7 | 1.10 (0.69 - 1.75) | 0.81 (0.49 - 1.35) | 0.59 (0.32 - 1.10) | 0.73 (0.31 - 1.73) | 0.50 (0.23 - 1.08) | 1.16 (0.68 - 1.98) |

*^*^ P<0.05 compared to reference group, ^†^ Asthma or wheeze or breathing difficulties,*  ^§^ *White UK = baseline,* *clustering in schools was accounted for using the xtlogit command in Stata with the random effect option*

Model 1: Adjusted for age

Model 2: Adjusted for (1) and disadvantage, maternal employment status, paternal employment status, family type, number of siblings

Model 3: Adjusted for (2) and generational status (pupil, parent and grandparent born abroad status)

Model 4: Adjusted for (3) and family history of asthma (parental and grandparental)

Model 5: Adjusted for (4) and parental smoking

Model 6: Adjusted for (5) and psychological well-being

Model 7: Adjusted for (6) and BMI percentile
